# ^36^Cl, a new tool to assess soil carbon dynamics

**DOI:** 10.1038/s41598-023-41555-x

**Published:** 2023-09-12

**Authors:** Cécile Grapeloup, Sophie Cornu, Xavier Giraud, Julie Pupier, Aster Team, Valery Guillou, Philippe Ciffroy, Beatriz Lourino Cabana, Cécile Couegnas, Christine Hatté, Lucilla Benedetti

**Affiliations:** 1 Aix Marseille University, CNRS, IRD, INRAE, Coll France, CEREGE, Aix en Provence, France; 2 EDF R&D, LNHE, 6 Quai Watier, 78400 Chatou, France; 3 LSCE, CEA, CNRS, UVSQ, Université Paris-Saclay, 91191 Gif-sur-Yvette Cedex, France; 4 Institute of Physics, Silesian University of Technology (SUT), 44-100 Gliwice, Poland

**Keywords:** Carbon cycle, Environmental impact

## Abstract

Soil organic carbon is one of the largest surface pools of carbon that humans can manage in order to partially mitigate annual anthropogenic CO_2_ emissions. A significant element to assess soil sequestration potential is the carbon age, which is evaluated by modelling or experimentally using carbon isotopes. Results, however, are not consistent. The ^14^C derived approach seems to overestimate by a factor of 6–10 the average carbon age in soils estimated by modeling and ^13^C approaches and thus the sequestration potential. A fully independent method is needed. The cosmogenic chlorine nuclide, ^36^Cl, is a potential alternative. ^36^Cl is a naturally occurring cosmogenic radionuclide with a production that increased by three orders of magnitude during nuclear bomb tests. Part of this production is retained by soil organic matter in organochloride form and hence acts as a tracer of the fate of soil organic carbon. We here quantify the fraction and the duration of ^36^Cl retained in the soil and we show that retention time increases with depth from 20 to 322 years, in agreement with both modelling and ^13^C-derived estimates. This work demonstrates that ^36^Cl retention duration can be a proxy for the age of soil organic carbon.

## Introduction

Soil organic carbon (SOC) is one of the largest surface pools of carbon^1^, with estimates ranging from 1500 to 2500 gigatons for the first meter of the soil^2^. This pool is one of the very few that humans can manage to partly mitigate annual anthropogenic CO_2_ emission (“4 per 1000”^3^ initiative launched by France during the COP21 based on a calculation made by Balesdent and Arrouays^4^. SOC can also foster food production and ecosystem stability^5^ can also be fostered. To achieve these multiple objectives, understanding SOC dynamics is crucial to unravel carbon soil sequestration potential^6^.

Soil organic matter is a complex mixture of molecules that evolve more or less rapidly towards mineralisation (CO_2_ emission) or towards more complex or stabilized molecules. The carbon sequestration potential of soil depends on the balance between SOC mineralization and stabilization over a longer or shorter period. Depending on the length of time that soil organic matter remains undecomposed, soil carbon is considered as falling into different pools. This is, however, only a conceptual framework of how SOC works. The estimation of the carbon content of these reservoirs is not achievable by experimentation and their modelling remains challenging. The representation of SOC in different pools is the basis used in Earth system models (ESMs)^7^. In order to validate this modelling approach, the SOC ages yielded by the models are compared to actual ages of SOC experimentally estimated from ^14^C^6,8^ and stable carbon isotope^9^ approaches. Meta-analyses using natural and nuclear bomb ^14^C peaks on soil profiles show that the mean derived-SOC ages for the first meter of the soil range from 3100 to 4800 ± 1800 years^6,8^. The ^14^C measured SOC age is more than six times higher than the SOC age yielded by the models (430 ± 50 years), resulting in a difference of 40 ± 27% in the soil’s potential to sequester atmospheric carbon^6^. On the other hand, Balesdent et al.^9^ estimated the SOC age distribution over the soil profile using the stable carbon isotopic signature. Their results provided a mean SOC age for the first meter of the soil of 489 ± 173 years, close to that yielded by ESM models^7^ but very different from those obtained by ^14^C dating^6,8^. The method used by Balesdent et al.^9^ is based on the proportion of new carbon atoms that was determined after a natural change in the stable carbon isotope signature of the vegetation at a known age. This approach is therefore not applicable everywhere. There is thus still a need to better assess the SOC age in order to discriminate between the two sets of SOC ages provided by ^14^C dating^6,8^ and C stable isotopes^9^.

Chlorine (Cl) is a highly mobile element that is slightly retained in soils in organochloride form^10^, with the notable exception of saline soils where Cl is also encountered as salt. The chlorination process occurs during the very first steps of soil organic matter degradation, mainly mediated by fungal activity^11,12^. The Cl forms covalent bonds as organic chloride compounds, of various high molecular weights^11^, that are then fragmented into various low molecular weight compounds. Laboratory experiments conducted on different soil samples evidenced a link between Cl chlorination and soil organic matter^13–17^. Although based on short-term laboratory experiments, these results, together with the fact that Cl is covalently bonded to organic compounds, suggest that organochlorine follows the same dynamics as soil organic matter, moving from one molecule to another through bioassimilation or being released by mineralization into the soil solution, when carbon returns to the atmosphere as CO_2_.

^36^Cl is a naturally occurring radionuclide (half-life: 301,000 year) formed in the atmosphere by spallation of ^40^Ar. Its production increased by three orders of magnitude above its natural level during the marine nuclear bomb tests that started in 1950, reached a peak in the early sixties, and lasted until the late 1970s^18–23^. Although stable chlorine isotopes (^35^Cl and ^37^Cl) have different sources, mainly produced by marine sprays, ^36^Cl follows the same biogeochemical cycle without isotopic fractionation^24^. Since organochlorine mimics the fate of the global soil organic matter, we propose that the fraction of ^36^Cl from the nuclear bomb tests retained in soils can be used to trace the SOC dynamics. This requires being able to quantify the fraction of ^36^Cl retained and the duration of ^36^Cl retention in soils.

## Results and discussion

### The fraction of ^36^Cl retained in soils is a function of the SOC content

**Figure 1:**
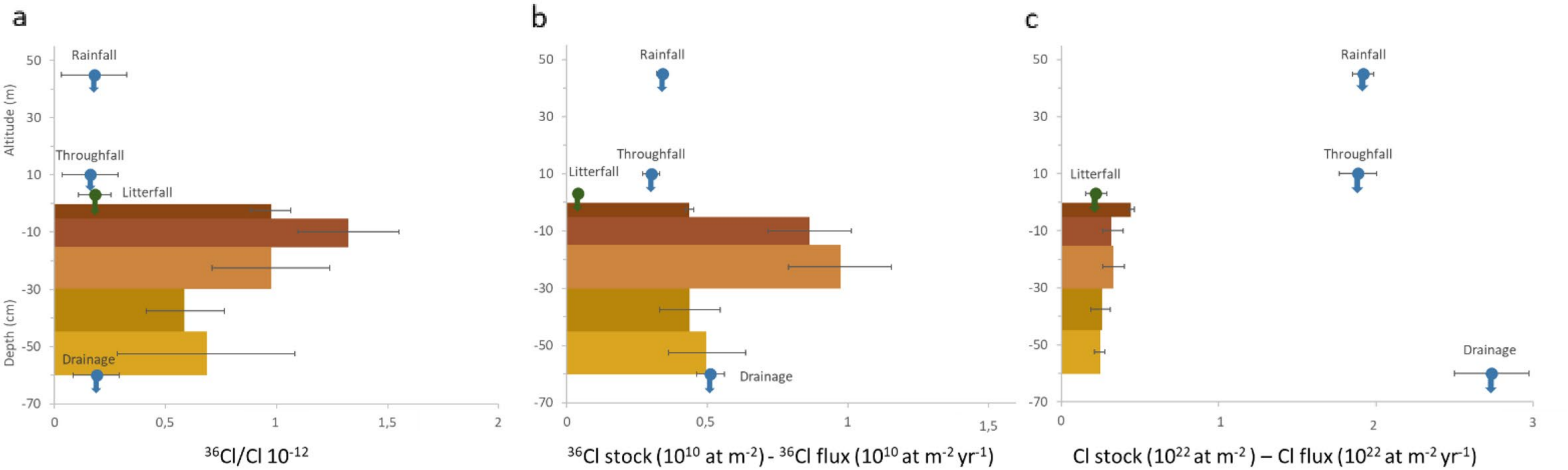
Measured ^36^Cl/Cl (unitless) (**a**), ^36^Cl (**b**) and Cl (**c**) stocks (bars, at m^−2^), water (blue dots) and litterfall (green dots) fluxes (at m^−2^ year^−1^). These fluxes were derived from measurements made in water (rainfall, throughfall—that is the water collected below the forest canopy—and soil water drainage at 60 cm depth) in 2012–2013, and stocks from soil sampled in 2010 along a soil depth profile at Montiers, France. Colours for the different soil stocks represent the different soil layers.

Batsviken et al.^15^ assessed experimentally, by incubating soil samples with a fixed amount of ^36^Cl, that 20% of ^36^Cl was retained in soils a few hours after injection but that only 4% remained after a few weeks. Gustavsson et al.^16^ suggested, also experimentally, that the amount of ^36^Cl retained in soils could vary 3- to 4-fold with the vegetation cover. However, the amount of ^36^Cl retained in soils under natural conditions has not yet been assessed. To fill this gap, we measured and modelled both Cl and ^36^Cl concentrations in a soil profile from a mature beech forest in Northern France. Figure 1 shows (1) the measured Cl and ^36^Cl inputs to the soil (by rainfall^25^, throughfall—portion of the rainfall that reaches the soil after penetrating the canopy—and litterfall) and outputs from the soil by soil water drainage in 2012–2013; (2) the Cl and ^36^Cl stocks in the different soil layers measured in 2010 and the corresponding ^36^Cl/Cl ratios (see “Methods”).

The ^36^Cl/Cl ratios measured in the annual rainfall and litterfall are not significantly different (Fig. 1a), inducing no isotopic fractionation against ^36^Cl by the vegetation. Cl is mainly in soluble anionic form in the leaves^24^, probably accumulated in the vacuole, and thus passively absorbed with the water flux by the plant, explaining the absence of isotopic fractionation. Since the ^36^Cl/Cl ratios of the input and output fluxes are not significantly different (Fig. 1a), we can also assume the absence of isotopic fractionation associated to the transfer of ^36^Cl through the soil and, as a corollary, a chlorination process also devoid of significant isotopic fractionation.

On the contrary, the ^36^Cl/Cl ratio measured in soil organic matter is two to six times higher than the ratio measured in the rainfall and litterfall and in drainage (Fig. 1a). The soil Cl and ^36^Cl stocks result from multi-annual accumulation and this result suggests that some of the retained ^36^Cl comes from the high ^36^Cl input due to nuclear tests. This also suggests that the retention duration of ^36^Cl at our site is higher than 40–60 years (i.e., duration estimated between the nuclear bomb testing period and the measurement dates).

The contribution of litterfall to soil ^36^Cl and Cl represents 11% of that due to rainfall (Fig. 1b,c). Throughfall Cl and ^36^Cl inputs equal rainfall Cl and ^36^Cl inputs. The ^36^Cl and Cl inputs to the soil over time can therefore be estimated on the sole basis of the ^36^Cl and Cl rainfall flux reconstruction.

**Figure 2:**
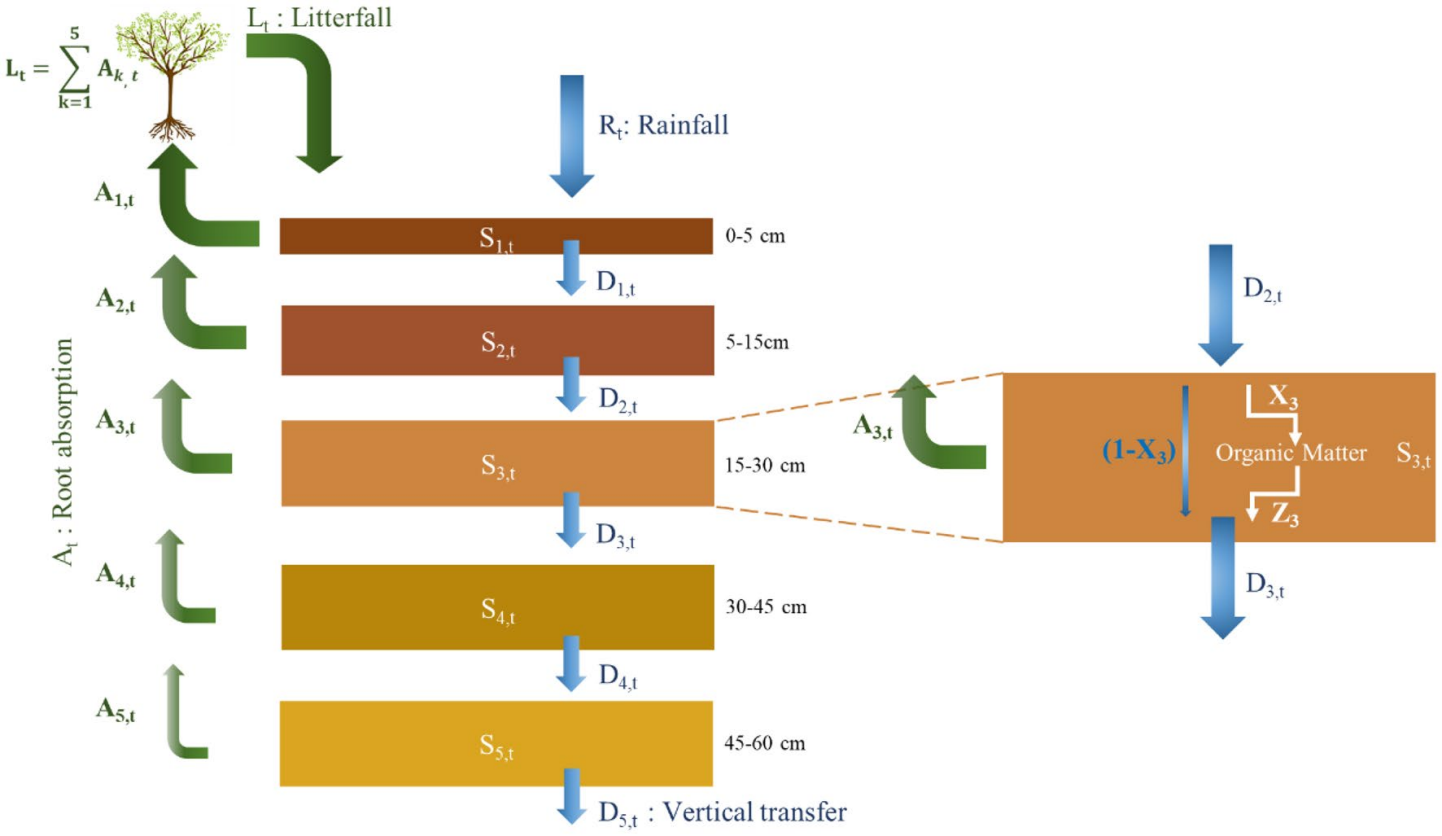
Schematic representation of the developed mass balance model. For each box (representing the different soil layers), input fluxes (I_k,t_) are rainfall (R_t_) and litterfall (L_t_) for the upper soil layer and, for the other layers, vertical transfer flux, mainly drainage, (D_k,t_) from the overlying layer. Outputs (O_k,t_) are vertical transfer (D_k,t_) and root absorption (A_k,t_) from the soil layer considered. Gas fluxes of Cl and ^36^Cl are neglected. At each time step, a fraction (X_k_) of the Cl and ^36^Cl input fluxes is retained in the soil layer k building the Cl and ^36^Cl stock (S_k,t_) while the remaining (1-X_k_) is lost from the soil by drainage. A fraction (Z_k_) of S_k,t_ is released in the soil solution and also drained. An annual time step (t) is used to smooth seasonal variations. For each soil layer k, S_k,t_ is calculated. The equations of the model are presented in “Methods” section. Colours for the different soil boxes represent the different soil layers. No humus layer is considered, as the residence time for Cl in the type of humus encountered in the studied site is less than a year, the time step of the model (see “Methods” section for further information).

We developed a model based on a mass balance calculation of the forest stocks and fluxes for each of the five soil layers (Fig. 2). A Monte Carlo approach was used to determine the fraction of the Cl and ^36^Cl input retained in the soil (X_k_) by adjusting output ^36^Cl stocks to the measured ones in 2010 (Fig. 2). Simulations were run from the pre-bomb period (1910 AD), when both Cl and ^36^Cl can be considered at steady state, up to 2020 AD. ^36^Cl anthropogenic production by nuclear bomb tests between 1952 and 1972^18^ was used as model input. Once the X_k_ had been determined, the Cl retention duration in the soil organic matter was calculated (for more details see “Methods”).

**Figure 3:**
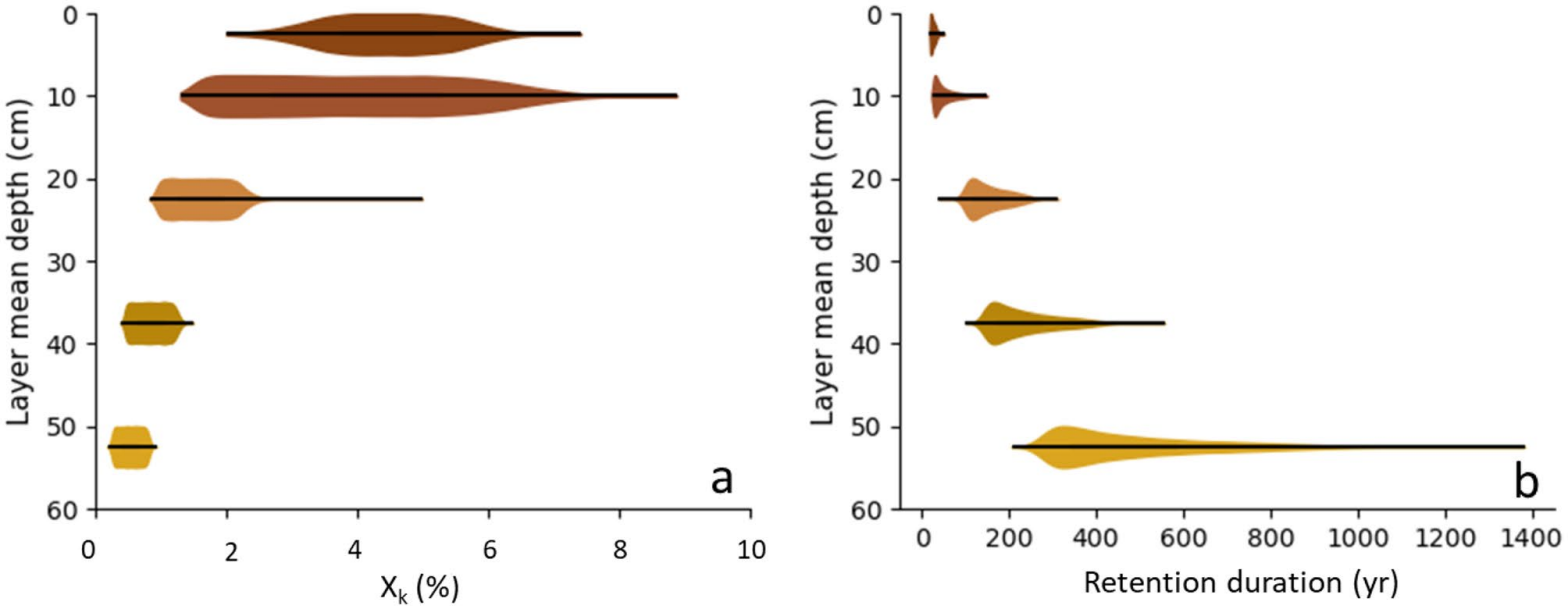
Depth evolution of the modelled probability density of: (**a**) the ^36^Cl fraction retained in the soil (X_k_); (**b**) the retention duration of ^36^Cl in the soil. The probability densities are obtained by running a few thousands of simulations, where every variable (rainfall, litterfall fluxes and the Cl and ^36^Cl stocks) is set randomly using a normal distribution defined by the measured variable values and their associated uncertainties. Colours represent the different soil layers. While the X_k_ distribution is normal, the retention duration distribution is log-normal (Supplementary data Fig. 1).

Figure 3 displays the depth-evolution of the X_k_ values, showing that they decreased exponentially, with a ninefold higher value at the surface than at depth. The associated uncertainties also decreased with depth. This result agrees with a high chlorination of organic matter, which is indeed classically observed in the upper soil layer^26,27^. The fraction of 4.5% yielded for the [0–5 cm] layer (X_1_) is in good agreement with the one obtained experimentally by Batskviken et al.^15^ (e.g., 4% after 4 months of incubation of topsoil samples). A more recent study^17^ reported X_k_ of the same order of magnitude for a forest soil with the same type of humus (mull). Lower X_k_ were however derived for forest soil associated with different types of humus layers (thicker: moder or mor). As in our results, the authors also observed a decrease in X_k_ with depth. Some much higher X_k_ were also observed in other studies (15–35%) with variable experimental conditions, and no clear drivers were found to explain these differences^16,28^.

**Figure 4:**
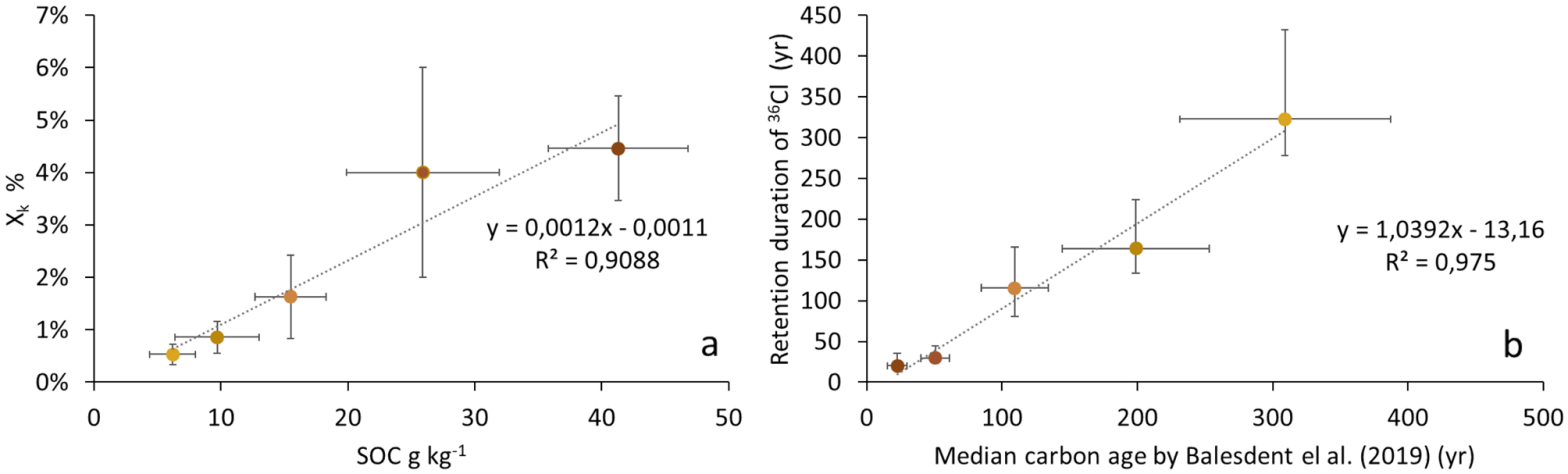
^36^Cl a tracer of the SOC. (**a**) The ^36^Cl fraction retained in the soil layers is a function of the SOC content. (**b**) The yielded retention duration of ^36^Cl in the soil is linearly correlated to the age of the SOC estimated by Balesdent et al.^9^ for tropical forest and pasture sites. Colours represent the different soil layers as reported in Figs. 1, 2 and 3.

The exponential decrease of X_k_ with depth (Fig. 3) is in good correspondence with the exponential decrease of SOC concentration with depth (Supplementary data Table 1) and the ^36^Cl fractions retained in the soil for the different soil layers are linearly correlated to SOC concentrations of these layers (Fig. 4a). This confirms that the dynamics of the ^36^Cl in organic form follows that of the soil organic matter.

### A ^36^Cl retention duration in soils equivalent to the age of the SOC

The retention duration of ^36^Cl in the SOC increased with depth from 20 ± 15 to 305 ± 110 years (Fig. 3b). The layer-by-layer ^36^Cl retention durations and the median SOC ages calculated by Balesdent et al.^9^ were of the same order of magnitude and the two datasets show a very nice correlation for all depths (Fig. 4b). While both methods obtained a SOC age of about 50 years for the first 0–30 cm of the soil, the SOC ages obtained from ^14^C measurements for the same depth were 1390 ± 310^8^. This discrepancy could be due to the presence in the soil of a very old fraction of SOC, even in topsoil, that no longer contributes to the active soil carbon cycle but contributes to the ^14^C age estimates^29^. Indeed, even in small proportions, old organic carbon leads to older average ages especially in the surface layers (0–30 cm) that are not representative of the real organic carbon dynamics of these layers^30^. The median SOC age obtained by Balesdent et al.^9^ provides more precise information on the dynamics of carbon in the different soil layers, while the ^14^C method makes it possible to estimate the proportion of very old organic carbon, part of which can be considered as inert. The ages obtained for ^36^Cl are very similar to the median ages obtained by the stable carbon isotope method^9^ and are therefore a good indicator of the active fraction of the SOC.

In conclusion, the ^36^Cl retention duration can be used as a proxy for the median age of active SOC, i.e., the reactive part of soil organic carbon. This age is also provided by the δ^13^C analysis, in the special cases of sites that have experienced a change in vegetation photosynthesis, at a known date^9^. Because it is not conditioned by this change, the ^36^Cl-based method may have greater potential for application. However, it has to be kept in mind that the present study is a proof of concept done on a single soil. It will have to be reproduced on other soil types: saline soil that requires adaptation of the model and other forest soils whose humus types could induce a variation in Cl and ^36^Cl cycles. Nevertheless, this method potentially provides strong constraints on the average age of reactive soil carbon that can be used by ESM models. In this light, the ^36^Cl method opens up a more accurate constraint on soil sequestration potential and a better assessment of anthropogenic carbon compensation by soils in the future.

## Methods

### Study site, flux and stock sampling

The experimental site consists in the 73 ha mature beech forest of the Perennial Environmental Observatory (PEO) located at Montiers-sur-Saulx, in the north-east of the Paris Basin, France (48° 31′ 54″ N, 5° 16′ 08″ E). The forest is a mature beech forest with a mean tree age of 50 yrs. It is part of a national forest, already recorded on the Cassini maps (XVIIth century), the Napoleonic land registry (1820–1866) and on old aerial photographs (1950–1965, Supplementary data Fig. 2). The soil is a Cambisol developed on Tithonian limestone. The humus of this forest soil was a mull.

A one-hectare station was established to monitor the following fluxes: rainfall, throughfall, drainage water at 60 cm depth and litterfall. Rainfall was sampled by open rainfall collectors located over the canopy as described by Pupier et al.^25^. Throughfall was collected monthly from March 2012 to February 2013 by four rain gutters placed 1 m above the forest floor and spatially distributed in order to capture the canopy variability and representing an equivalent surface of 0.39 m^2^. The water drainage at 60 cm depth was collected with the same frequency and time period by three lysimetric plates measuring 0.12 m^2^. This device only collects soil gravity water. An average sample of 1 L was made on site from these three lysimetric plates. All the devices were of high density polyethylene (HDPE). In order to avoid possible contamination, all the material was washed with ultra-pure water.

The litterfall was collected over the year 2012 thanks to 6 L trays of 1 m^2^ to capture the canopy variability. The litter was weighed, dried at 65 °C in a ventilated oven for 1 week and ground in a ring mill. An annual composite sample was created.

The soil was sampled by coring in 2010. The following depth intervals were sub-sampled: 0–5 cm, 5–15 cm, 15–30 cm, 30–45 cm and 45–60 cm. Three cores were sampled and mixed to form an average sample. The soil samples were dried at 35 °C and sieved at 2 mm.

### Cl and ^36^Cl extraction and analysis

The Cl and the ^36^Cl in the solid matrices were extracted by hydropyrolysis and trapped in ultrapure water after a protocol adapted from Cornett et al.^31^ and Herod et al.^32^. A selective extraction of soluble Cl and ^36^Cl was also performed by equilibrating 5 g of dry soil with 40 mL of ultra-pure water for an hour.

The Cl and ^36^Cl in the liquid samples were precipitated as AgCl by adding nitric acid and silver nitrate following a standard procedure as described in Pupier et al.^25^ and Bouchez et al.^33^. ^36^Cl and Cl concentrations were measured by isotope dilution accelerator mass spectrometry at ASTER-CEREGE^34,35^ (Accelerator for Earth Sciences, Environment, Risks). Both the ^36^Cl/^35^Cl and the ^35^Cl/^37^Cl ratios were measured and normalized to the in-house standard SM-CL-12 (^36^Cl/^35^Cl value of (1.428 ± 0.015) × 10^–12^), assuming a natural ^35^Cl/^37^Cl ratio of 3.127.

### Cl and ^36^Cl flux and stock calculation

While both soluble and total ^36^Cl and Cl concentrations were measured, we decided to calculate total stocks that were considered as organic as:

1. soluble fractions were very small, especially for ^36^Cl (one order of magnitude lower), compared to the total fraction;
2. the considered soil does not contain salts
3. therefore, the soluble Cl and ^36^Cl can either come from the Cl and ^36^Cl contained in the soil water at the sampling time, or from Cl and ^36^Cl released in solution from the organic matter at the extraction time. The concentrations recorded in the soil water were too low to explain the Cl and ^36^Cl extracted experimentally^36^. We therefore considered that the soluble Cl and ^36^Cl were organically bound Cl and ^36^Cl

The ^36^Cl and Cl stocks (S_k,2010_; at m^−2^) were calculated using the ^36^Cl and Cl concentrations (C _k,2010_; at g^−1^), the soil layer bulk density (ρ_k_; g cm^−3^) and the soil layer thickness (d_k_; cm) according to Eq. (1):

1 $${S}_{k,2010}={\complement }_{k,2010} {\rho }_{k} { d}_{k} {10}^{4}$$

The calculated stocks are reported in Supplementary data Table 2.

The fluxes (F; at m^−2^ year^−1^) were calculated using the ^36^Cl and Cl concentrations (C; at g^−1^) and the measured annual litterfall/water flux (Φ) (Supplementary data Table 3) according to Eq. (2):

2 $$F=C\Phi $$

No Cl and ^36^Cl stock in humus was considered as the considered forest has a mull type of humus, that is a very thin and reactive humus. Dincher^37^ demonstrated for the same site that highly soluble elements were lost from the litter in less than a year. Since Pupier^36^ showed that most Cl and ^36^Cl in litter was in soluble form, we considered that all of it was lost to the soil within the year, the time step of our model, and hence did not consider a humus compartment. This assumption was confirmed experimentally for a mull by Svensson et al.^14^.

The uncertainties on the stocks and fluxes were calculated based on classical uncertainty propagation equations considering: (1) the analytical uncertainties for the ^36^Cl and Cl concentrations; (2) 5% of uncertainty for bulk density; (3) 10% for the layer thickness, the litterfall mass and the water drainage volume; and (4) 3% uncertainty for rainfall as suggested by Météo France specifications.

### Modelling approach

### Description of the model

The model is a mass-balance approach that considers stocks and fluxes of Cl and ^36^Cl in the different soil layers (Fig. 2).

For each box, the mass balance is evaluated at an annual time step ($\Delta t$ is set to 1 year) to smooth seasonal variations, and the stock of year *t*, for layer *k,* S_k,t_, is described by Eqs. (3) and (4):

3 $${S}_{k,t}=\left(1-{Z}_{k}\right) {S}_{k,t-1}+{X}_{k} {I}_{k,t} \Delta t$$

4 $${D}_{k,t}=\left(1-{X}_{k}\right) {I}_{k,t}+\frac{{Z}_{k} {S}_{k,t-1}}{\Delta t}-{A}_{k,t}$$

with

$${I}_{1,t}= {L}_{t}+ {R}_{t}$$

$${I}_{k,t}= {D}_{k-1,t}\mathrm{ \,for \,k}>1$$

where S_k,t−1_ is the standing stock of the previous year on the same layer *k*. The input fluxes (I_k,t_) for each layer come from the vertical transfer flux (D_k−1,t_) from the overlying compartment (layer k − 1), except for the upper soil layer which receives rainfall (R_t_) and litterfall (L_t_). X_k_ is the fraction of the Cl and ^36^Cl input that is retained in the soil at each time step. Z_k_ is the fraction of the preexisting stock that is released.

Applied to a mature forest, Cl is considered at steady state^38^ so that the total annual root absorption over the different layers is assumed to equal the annual litter fall (L_t_) that corresponds to the annual litter production.

5 $${L}_{t}=\sum \limits_{k=1}^{5}{A}_{k,t}$$

As the root distribution is classically considered as exponentially decreasing in depth^39^, we assumed an exponential decrease for the root absorption depth distribution. Other root absorption depth distributions were tested. They had no impact on the model results.

Stocks (S_k,t_) in ^36^Cl are expressed in at m^−2^ and ^36^Cl fluxes (I_k,t_, D_k−1,t_, L_t_, R_t_) in at m^−2^ year^−1^, so that X_k_ and Z_k_ are in %.

In addition in a system at steady state, a residence time of Cl (T_R,k_) can be calculated for each soil layer according to Eq. (6):

6 $${T}_{R,k}=\frac{{S}_{k,t}}{{I}_{k,t}}$$

Using residence time reduces the system of equations by expressing Z_k_ as a function of X_k_ (by combining Eq. (6) with Eqs. (3) and (4)), as in Eq. (7).

7 $${Z}_{k}=\frac{{X}_{k} \Delta t}{{T}_{R,k}}$$

As a result, the model depends on the initial stocks (of Cl and ^36^Cl), the history of the input fluxes (litterfall and rainfall) and only one parameter, the fraction X_k_.

Since the Cl stock in the layer k is considered at equilibrium and is yearly fed by a fraction of the Cl input (X_k_·I_k,t_,) fixed on organic matter, the retention duration of Cl in organic matter in each layer can be calculated as in Eq. (8):

8 $${T{\prime}}_{R,k}=\frac{{S}_{k,t}}{{X}_{k} {I}_{k,t}}$$

### Initial state and forcing scenario

Since the system is considered at steady state for Cl, the soil Cl stock is constant in time and thus the initial stock of Cl in each layer is known. The residence times (T_R,k_) of Cl in the different soil layers can therefore be calculated.

Before the nuclear tests, the ^36^Cl input to the ecosystem came only from the cosmogenic source and is considered stable in time. At that period the system can thus be considered at steady state for ^36^Cl. Since there is no isotopic fractionation for Cl in the environment, the residence time of the two elements in soil layers are considered equal and the initial ^36^Cl stock for the different soil layers S_k,0_ can be calculated (Eq. 9)

9 $${S}_{k,0}{(}^{36}Cl)={I}_{k,2012}{(}^{36}Cl) {T}_{R,k}(Cl)$$

A forcing scenario is used that consists in the ^36^Cl rainfall input from the pre-bomb period (1940) to year 2020 (Supplementary data Fig. 3). The natural background cosmogenic ^36^Cl input is considered as constant in time and equal to the one measured on the studied site in 2012–2013; the anthropogenic ^36^Cl produced by nuclear bomb tests from 1952 to 1972 was determined on the basis of the ECHAM5-HAM general circulation model simulation of Heikkila et al.^18^. Litterfall accounts for 11% of the Cl isotope rainfall inputs (Fig. 1, Supplementary data Table 3).

### Testing parameters and model-data comparison

A simulation consists in running the yearly mass balance from the pre-bomb period to the year 2020 using the ^36^Cl precipitation history for the rainfall (see previous section) for a considered X_k_ value chosen between 0 and 100%. Only the X_k_ values that provided a ^36^Cl stock that was not significantly different, for a given soil layer, from the measured one (within measurement uncertainty) were retained.

For each soil layer, several X_k_ values can be used to fit the actual ^36^Cl soil stocks. These values range from a few percent to ≈ 30% (Supplementary data Fig. 4). Since measured Cl and ^36^Cl input and output fluxes (Fig. 1) suggested a low fraction of Cl and ^36^Cl retained in the soil, we considered only the lowest X_k_ values.

All previous variables (rainfall, litterfall, Cl and ^36^Cl stocks and fluxes) come with uncertainties. These uncertainties were considered by running ten thousand simulations, where every variable was set randomly using a normal distribution defined by its measured values and associated uncertainties. The tested values of X_k_ were chosen randomly with a homogeneous distribution (between 0 and 1).

X_k_ and Z_k_ are fractions. For clarity's sake, they were multiplied by 100 so as to be presented as percentages in the figures.

### Supplementary Information

Supplementary Information.

## Data Availability

All data generated or analysed during this study are included in this published article and its supplementary material file.
