# Supplementary material for: 36Cl, a new tool to assess soil carbon dynamics
Source: Sci Rep. 2023 Sep 12;13:15085. doi: 10.1038/s41598-023-41555-x (PMC10497631; doi:10.1038/s41598-023-41555-x)

**Supplementary Material**


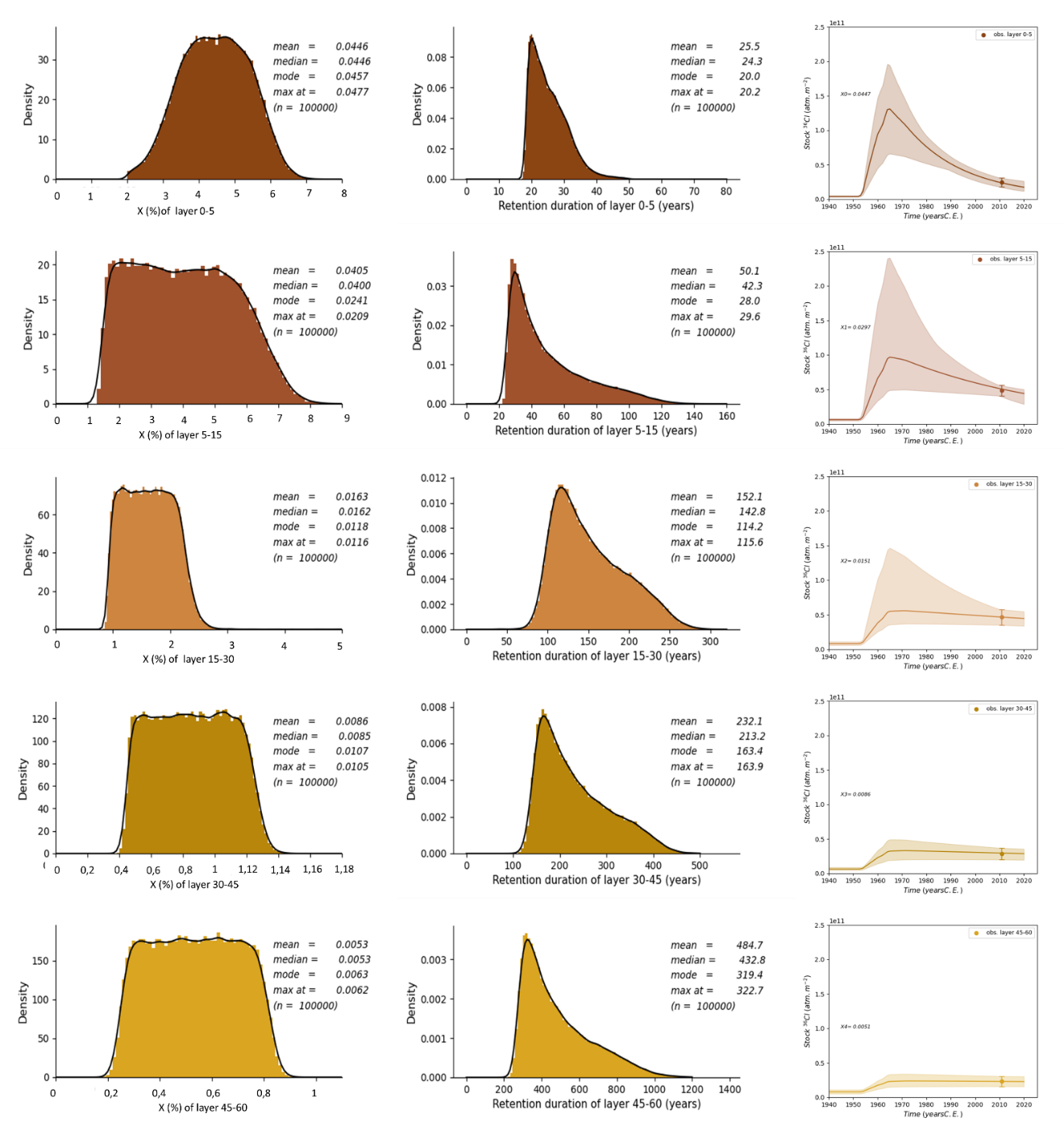


**Supplementary data Figure 1 | Modelled probability density of the ^36^Cl fraction (X_k_) retained in the different soil layers (left column), retention duration (middle column) and stocks building along time with measured stocks (right column).**

**
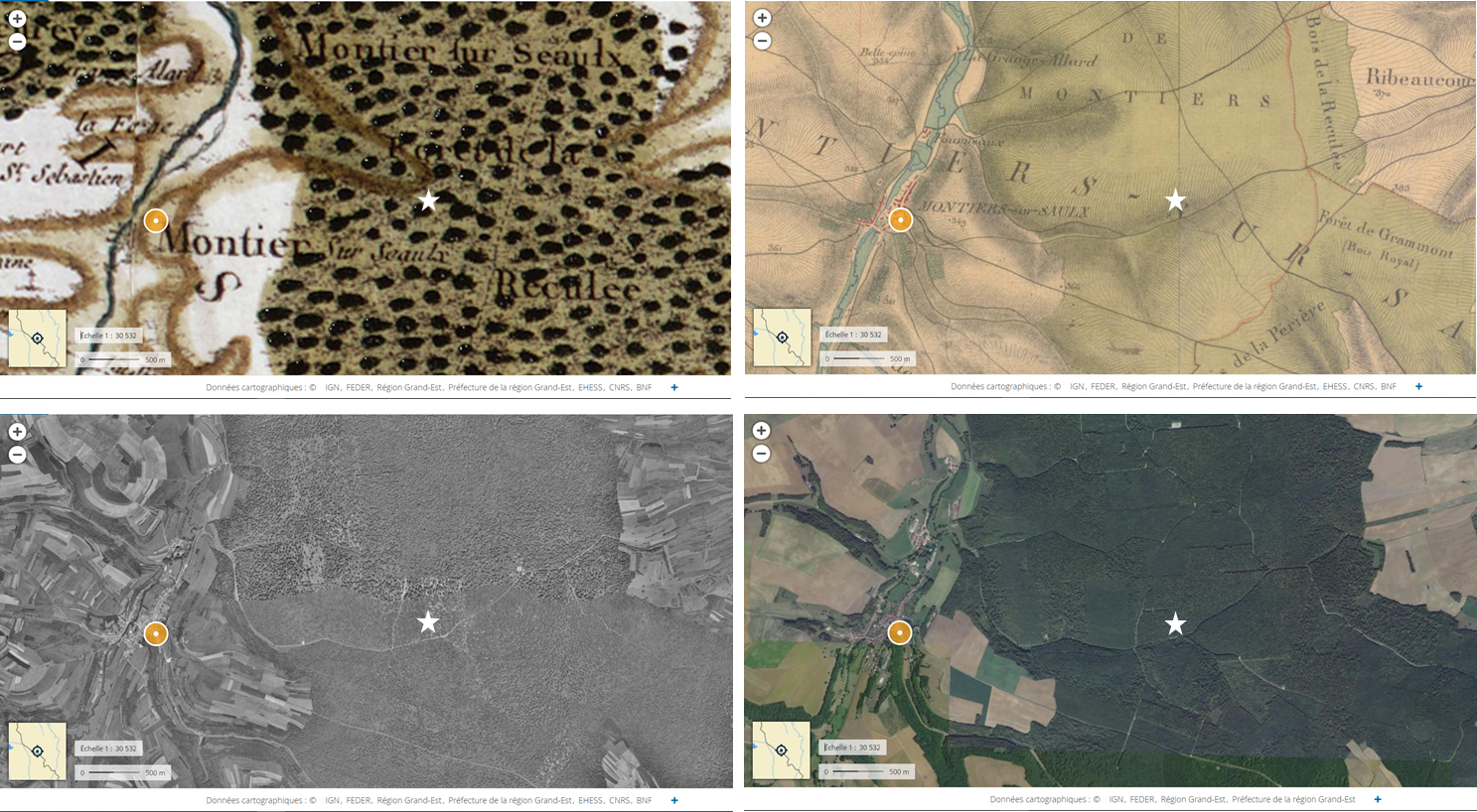
**

**Supplementary data Figure 2 | Forest sampled along time: a- Cassini map (XVIIth century), b- Napoleonic cadastre (1820-1866), c- aerial photograph (1950-1965), d- aerial photograph (2018). The white star represent the sampling site. Maps and aerial photographs are from the IGN site (**<https://www.geoportail.gouv.fr/carte>**, accessed on the 1st of February 2023.**

**
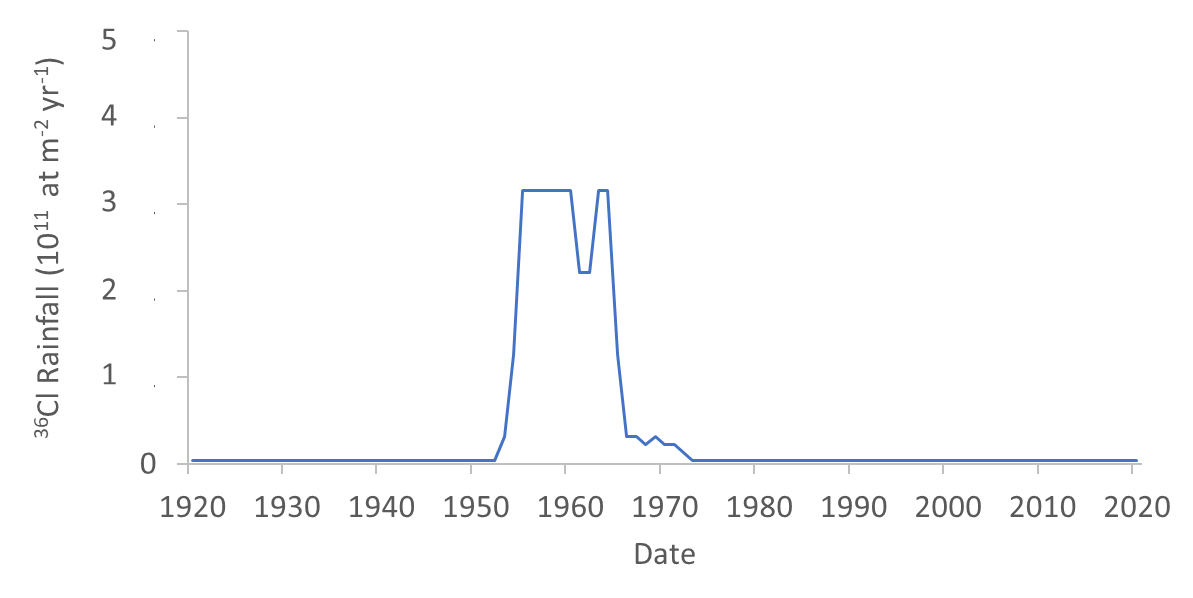
**

**Supplementary data Figure 3 | ^36^Cl rainfall input from the pre-bomb period (1940) to 2020.**

**
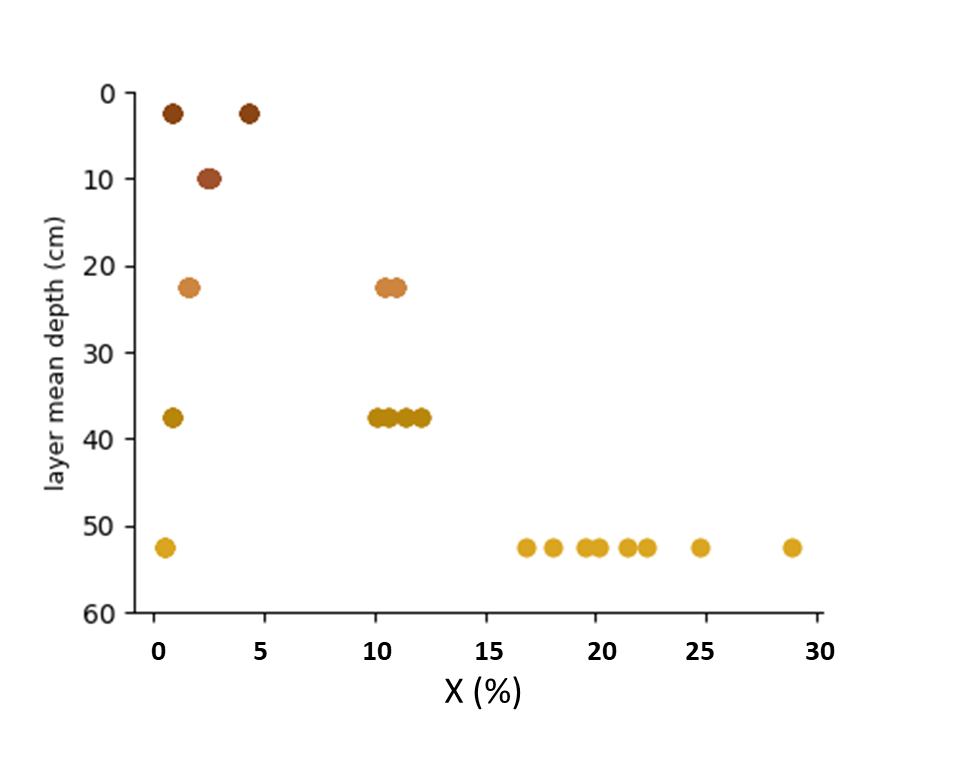
**

**Supplementary data Figure 4 | X_k_ values fitting the actual ^36^Cl stocks for the different soil layers.**

**Supplementary data Table 1: Main soil characteristics of the Montiers forest site.**


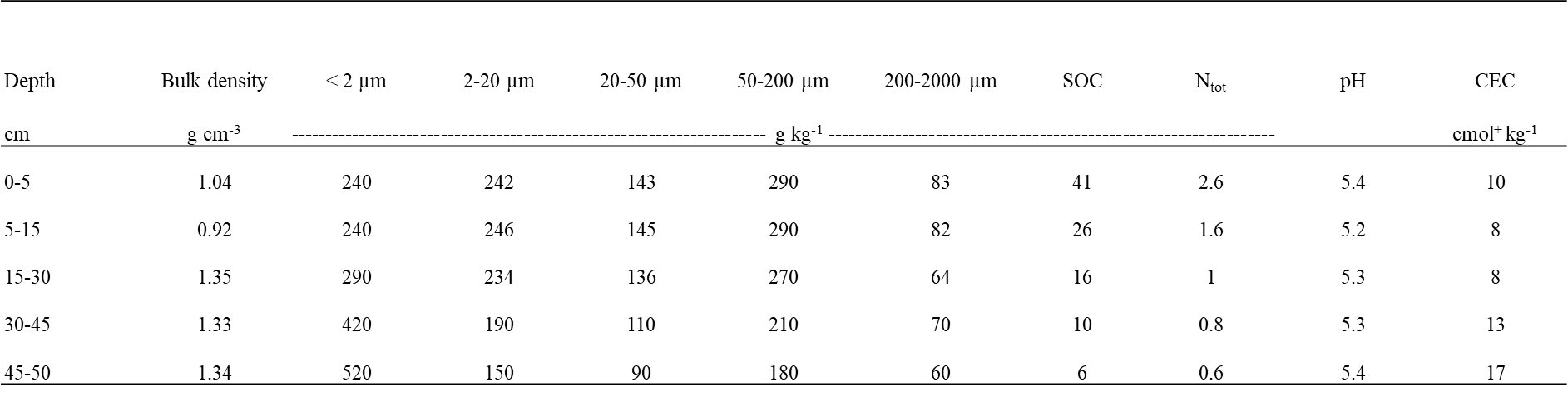


**Supplementary data Table 2: Cl and ^36^Cl soil concentrations and stocks of the Montiers forest site.**

**
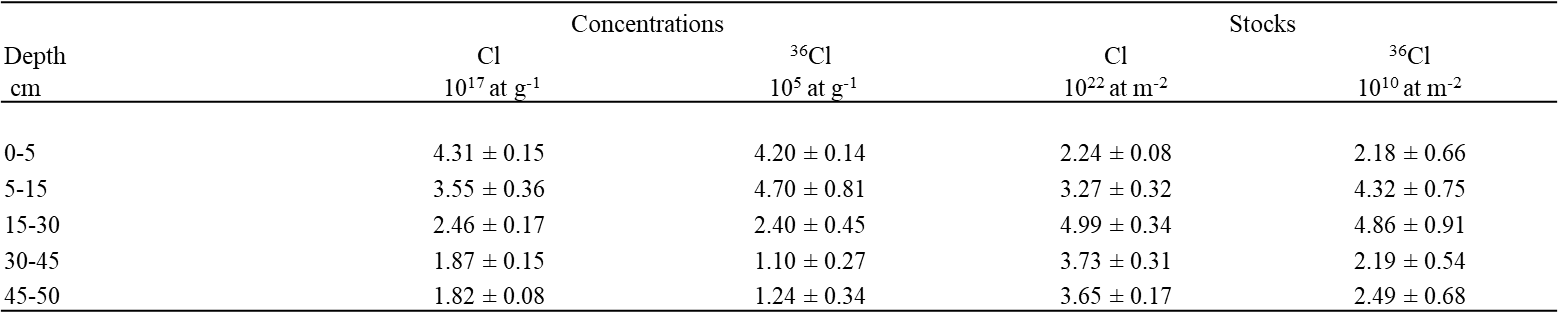
**

**
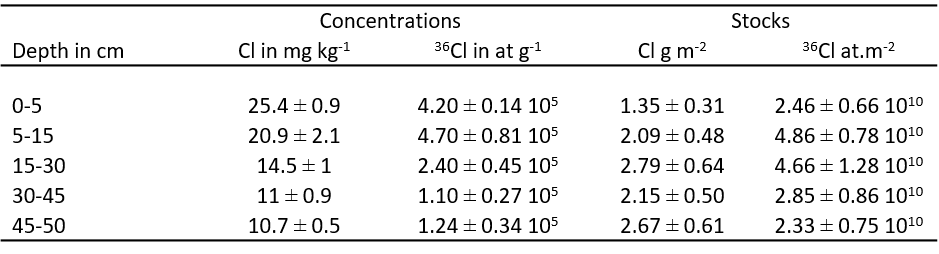
 Supplementary data Table 3: Cl and ^36^Cl concentrations and flux of the Montiers forest site.**


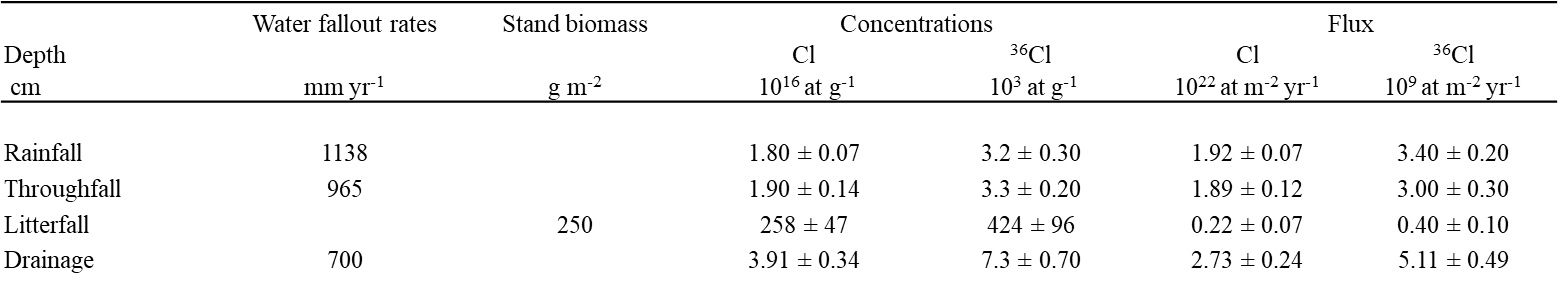

Supplement: Supplementary file 1 — Supplementary Information. [file 41598_2023_41555_MOESM1_ESM.docx]
